# The opportunities and obstacles of studying telomere length as a biological aging marker in psychiatry

**DOI:** 10.1192/j.eurpsy.2021.195

**Published:** 2021-08-13

**Authors:** J. Verhoeven, B. Penninx

**Affiliations:** Department Of Psychiatry, Amsterdam University Medical Centers, Vrije Universiteit and GGZ inGeest, Amsterdam, Netherlands

**Keywords:** Biology, Aging, telomere, Depression

## Abstract

Aging can be described as the life-long accumulation of damage to the tissues, cells, and molecules of the body. One of the most widely used markers to study biological aging is telomere length. Telomeres are non-coding DNA structures located at the ends of chromosomes that become progressively shorter with age. Research in the past decade showed that persons with psychiatric disorders such as major depressive disorder, anxiety disorder or posttraumatic stress disorder on average have shorter telomeres, which might help explain the high levels of somatic morbidity in these patients. While telomere length is an elegant aging biomarker, reflecting a biological process in most living species, there are also some challenges. In human studies, the between-person variation is large and shortened telomeres showed not to be specific to psychiatric diagnosis but rather to a multitude of psychological and physiological stressors. Telomere length might therefore not be a diagnostic marker. It could, nonetheless, be an interesting target for pharmacological, psychological or exercise treatment. If persons with psychiatric disorders age biologically faster, to what extend can this be process be halted or even reversed with successful treatment? Other opportunities and obstacles of studying telomere length as a biological aging marker in psychiatry will be discussed in this session.

**Disclosure:**

No significant relationships.

